# The Old and New Testaments of gene regulation: Evolution of multi-subunit RNA polymerases and co-evolution of eukaryote complexity with the RNAP II CTD

**DOI:** 10.4161/trns.28674

**Published:** 2014-04-07

**Authors:** Zachary F Burton

**Affiliations:** Department of Biochemistry and Molecular Biology; Michigan State University; East Lansing, MI USA

**Keywords:** Chromatin and transcription, Eukaryotic transcription, Prokaryotic transcription, RNA polymerases, transcriptional elongation, transcriptional initiation, transcriptional termination

## Abstract

I relate a story of genesis told from the point of view of multi-subunit RNA polymerases (RNAPs) including an Old Testament (core RNAP motifs in all cellular life) and a New Testament (the RNAP II heptad repeat carboxy terminal domain (CTD) and CTD interactome in eukarya). The Old Testament: at their active site, one class of eukaryotic interfering RNAP and ubiquitous multi-subunit RNAPs each have two-double psi β barrel (DPBB) motifs (a distinct pattern for compact 6-β sheet barrels). Between β sheets 2 and 3 of the β subunit type DPBB of all multi-subunit RNAPs is a sandwich barrel hybrid motif (SBHM) that interacts with conserved initiation and elongation factors required to utilize a DNA template. Analysis of RNAP core protein motifs, therefore, indicates that RNAP evolution can be traced from the RNA-protein world to LUCA (the last universal common ancestor) branching to LECA (the last eukaryotic common ancestor) and to the present day, spanning about 4 billion years. The New Testament: in the eukaryotic lineage, I posit that splitting RNAP functions into RNAPs I, II and III and innovations developed around the CTD heptad repeat of RNAP II and the extensive CTD interactome helps to describe how greater structural, cell cycle, epigenetic and signaling complexity co-evolved in eukaryotes relative to eubacteria and archaea.

Multi-subunit RNA polymerases (RNAPs) synthesize a RNA polymer from a DNA template.^1^^-^^3^ Figure 1 shows a partial RNAP image emphasizing the active site, which is wedged between two-double psi β barrels (DPBBs) and the long (~40 amino acid) bridge α-helix and the mobile trigger loop. Because RNAPs translocate (move) along a DNA template in single base steps to form the RNA polymer, RNAPs are referred to as “molecular motors.” The catalytic mechanism involves two clustered magnesium (Mg) atoms. Polymerization requires a nucleoside triphosphate (NTP) substrate (ATP, GTP, CTP or UTP) from which a nucleoside monophosphate (NMP) unit is added to the growing RNA polymer. The NTP substrate is specified by Watson-Crick base pairing to the DNA template strand. Because these RNAPs are dependent on a DNA template, they are considered to be DNA-dependent RNA polymerases or DDRPs.

**Figure:**
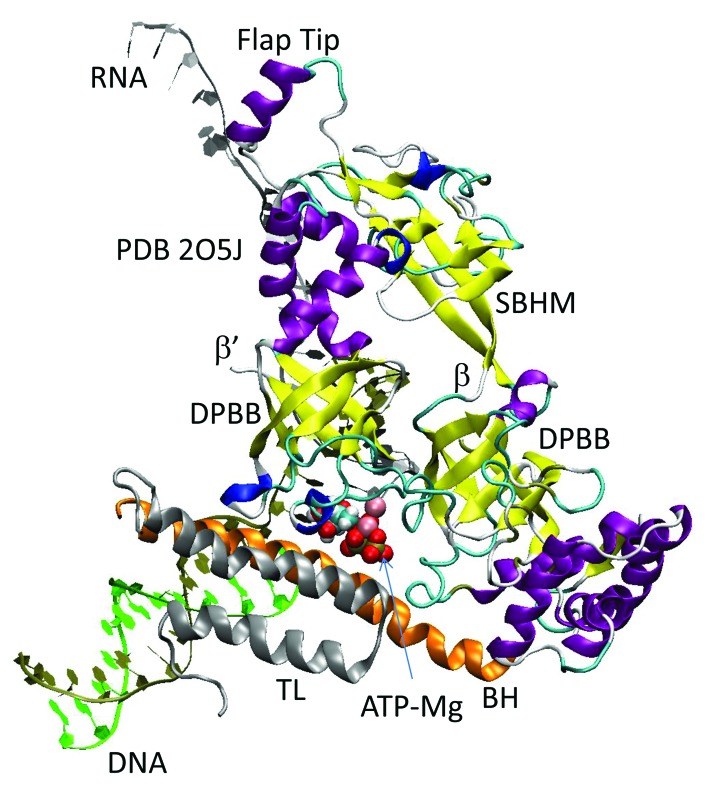
**Figure 1.** The active site of eubacterial Tt RNAP. The catalytic center is occupied by ATP-Mg (space-filling representation). β sheets are yellow. RNA is silver. DNA is gold (template strand) and green (non-template strand). The active site is wedged between the closed trigger loop (TL; silver), bridge helix (BH; orange) and two DPBBs. This image was drawn using PDB 205J^42^ with the program Visual Molecular Dynamics (used for all molecular graphics).^43^

Multi-subunit RNAPs are found in all cellular life on earth, which has diverged into three domains: eubacteria, archaea and eukarya (including humans). Eubacteria and archaea have a single RNAP. Within the cell nucleus, eukarya have at least three: RNAP I, II and III, allowing for much broader specialization in gene regulation. RNAP I synthesizes rRNA. RNAP II synthesizes mRNA, which specifies protein sequences. RNAP III synthesizes 5S RNA, tRNA and some small RNAs. RNAP IV and V are found in some plants and synthesize regulatory RNAs. That highly homologous multi-subunit RNAPs are found in all cellular life is evidence for a last universal common ancestor (LUCA) from which eubacteria, archaea and eukarya are derived, making *Escherichia coli* (eubacteria) and humans (eukarya) distant cousins. Analysis of RNAP structures shows significant family resemblance between eubacteria and eukarya (see below). Of course, this argument for LUCA and the relatedness of extant life forms could be advanced based on the highly conserved structures of many, many proteins, not just RNAP. LUCA is ancient: approximately 3.5 to 3.8 *billion* years ago (bya). With respect to many core protein motifs in essential processes, there appears to be surprisingly little evolutionary innovation since LUCA (see below).^4^

## The Old Testament

Multi-subunit RNAPs evolved around two DPBBs (Figs. 1 and 2).^4^^-^^6^ Remarkably, the two DPBBs are found within the two largest RNAP subunits (Fig. 2A), which were not previously known to be related based on simple amino acid sequence comparison.^4^ DPBBs are a barrel shape formed from 6-β sheets with the specific order and polarity shown in Figure 2. β sheets assemble in a parallel or anti-parallel orientation and neighboring sheets are held together by hydrogen bonds. Because both the β type and β’ type RNAP subunits (using eubacterial RNAP nomenclature as I do throughout) have structurally related DPBBs, the β and β’ subunits of RNAP are considered to be genetically related to one another.^4^ For comparison, Figure 2B shows the related two DPBB active site structure of a RNA-dependent RNA polymerase (RDRP) that synthesizes interfering RNA in the eukaryote *Neurospora crassa*.^7^ In this case, both DPBBs are within a single ββ’ type peptide chain. Because both DDRPs and RDRPs can be of the ββ’ two-DPBB type, DDRPs appear to be evolved from RDRPs and, therefore, appear to be rooted in the RNA-protein world (compare Figures 2A and 2B).^4^ The two-DPBB type RDRP (Fig. 2B) has been lost in vertebrates (including humans), insects and some yeast and is not present in eubacteria or archaea. DPBB-type RDRPs can be substituted by non-homologous or two-DPBB type RNAPs that synthesize short regulatory RNAs, making two-DPBB type RDRPs potentially redundant and replaceable in evolution.^4^ At the Mg binding site, just N-terminal to the β’ subunit β6 sheet, two-DPBB type RDRPs have the amino acid sequence DbDGD (D = aspartic acid; b = a bulky amino acid; G = glycine). Most DDRPs have the homologous amino acid sequence NADFDGD (N = asparagine; A = alanine; F = phenylalanine, which is a bulky amino acid). Mg (with charge +2) is held by the 3 Ds (aspartic acid (charge -1 for each D)). Note that the β and β’ type DPBBs of DDRPs and RDRPs are related to one another by simple translation with very little rotation. Aspartic acid residues that interact with Mg from the β DPBB (ED; just before β2) and the β’ DPBB (NADFDGD; just before β6) are not related sequences and are located in different segments of the barrels.

**Figure:**
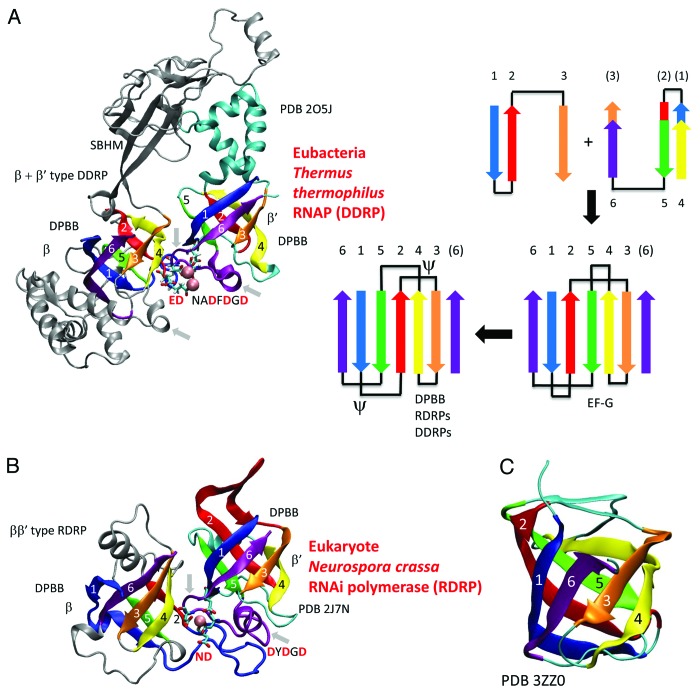
**Figure 2.** Two-DPBB type DDRPs and RDRPs. (**A**) Tt RNAP DPBBs. (**B**) *Neurospora crassa* interfering RNA polymerase (RDRP) DPBBs. (**C**) A simpler 6-β sheet barrel from translation elongation factor EF-G from *Staphylococcus aureus* from which a DPBB can be derived via β sheet exchange (β2 for β5). The schematic indicates a potential two step evolution of DPBBs by duplication of a simple 3-sheet motif to form a simple 6-sheet barrel (as in EF-G) followed by switching the order of β2 and β5. The color coding for the β sheets is indicated, and sheets are numbered β1→β6 according to their order in the peptide chain. The Greek letter psi (ψ) indicates the two psi pattern in the DPBB. Connections in the β type DPBB are silver; connections in the β’ type DPBB are cyan; Mg is magenta. Active site aspartates are shown. Small silver arrows emphasize a conserved loop (Mg binding) and motif between β5 and β6.

The schematic shown in Figure 2 indicates a two-step model for evolution of the DPBB motif, starting with a common 3-antiparallel β sheet motif with a space separating β2 and β3. Duplication of the 3-β sheet motif can result in formation of a 6-β sheet barrel, with all 6 antiparallel nearest neighbor β sheets, as in translation elongation factor and mRNA translocating GTPase EF-G (Fig. 2C). By switching the positions of β2 and β5 in the barrel, the DPBB motif is formed with neighboring sheets β1 and β5 parallel and β2 and β4 parallel (Figs. 2A and 2B and schematic). DPBBs are named for the two Greek letter psi (ψ) patterns formed by crossing peptide chains in forming the barrel (see Figure 2 schematic).^4^^,^^8^

### Conserved initiation and elongation factors and the SBHM

The sandwich barrel hybrid motif (SBHM) embedded between the 2nd and 3rd β sheets of the β subunit type DPBB provides an interaction surface for initiation and elongation factors that are conserved in the three domains of life (Fig. 3).^6^ In eubacteria, sigma factors allow specific initiation from a DNA template. Sigma factors are related to TFB in archaea and TFIIB (RNAP II) and Brf-1 (RNAP III) in eukarya, factors that aid accurate initiation (two helix-turn-helix motifs are conserved) (http://jivarahasya.blogspot.com/).^6^ In eubacteria, during RNA polymer elongation, NusG factors bump sigma off of the SBHM. Remarkably, NusG relates to Spt5 in archaea and eukarya. Therefore, the machinery that allows use of a DNA template for initiation and elongation of transcription and its SBHM interaction surface on RNAP are conserved in evolution. This is remarkable preservation of interdependent and interacting protein structures and functions (Fig. 3). Figure 4 shows that yeast (Sc) RNAP II and eubacterial *Thermus thermophilus* (Tt) RNAP are highly conserved in core motifs, demonstrating their kinship.

**Figure:**
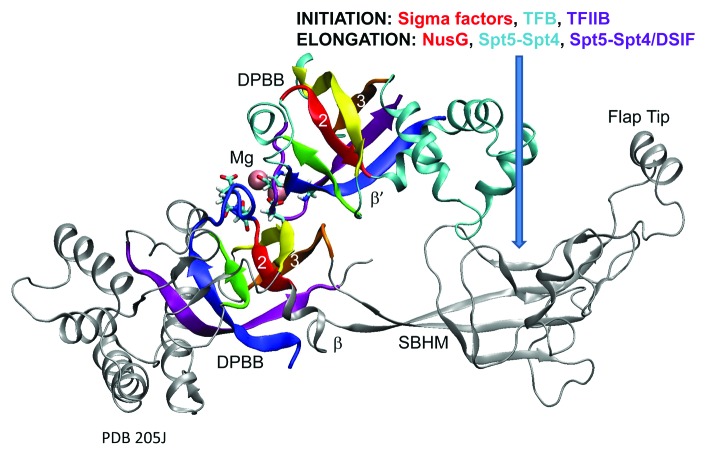
**Figure 3.** A SBHM necessary to utilize a DNA template. The SBHM is inserted between β2 and β3 of the β subunit type DPBB and is a landing pad for conserved initiation and elongation factors in the three domains of cellular life: eubacteria (red), archaea (cyan), eukarya (purple). Coloring of the DPBBs is as in Figure 2.

**Figure:**
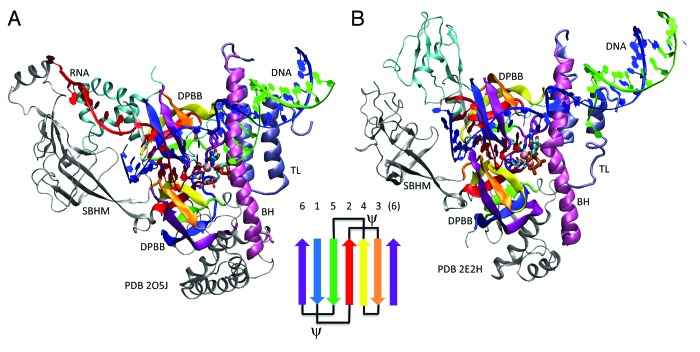
**Figure 4.** Multi-subunit RNAPs from eukarya and eubacteria are closely related. (**A**) Sc RNAP II (eukaryotic). (**B**) Tt RNAP (eubacterial). DPBBs are colored as in Figure 2 (see schematic). Only a subset of core motifs is shown. RNA is red; DNA is blue (template strand) or green (non-template strand). The bridge helix (BH) is magenta; the trigger loop (TL) is ice blue.

### The RNA-protein world

The concept of the RNA world is that, sometime prior to LUCA, RNA comprised genomes rather than DNA. Progeny derived from LUCA are DNA organisms, so LUCA must also have been a DNA organism.^4^ In the RNA (or RNA-protein) world, many or most enzymes were thought initially to be ribozymes: catalytic RNA molecules. In the modern day, ribosomes, which synthesize proteins (polymers of amino acids) reading a template mRNA and using tRNAs and rRNAs, are considered to be ribozymes because rRNA provides polypeptide synthesis function. Ribonuclease P is another ribozyme that has persisted through evolution. Ribosomes and ribonuclease P include protein components, but the protein co-factors do not participate directly in catalysis. It is thought that most ancient ribozymes were invaded by protein co-factors that eventually took over RNA catalytic function to evolve into modern protein enzymes, most of which eventually shed RNA components.^4^ A fascinating idea about ribosomes is that they remained ribozymes because protein catalysts are less efficient at building peptide chains than the RNA enzyme that has, therefore, persisted in evolution (http://jivarahasya.blogspot.com/). So the ribosome ribozyme function was selected in competition with less capable protein functions. By contrast, proteins are adept at binding positively charged Mg atoms through negatively charged aspartic acid, as in RNA synthesis by multi-subunit RNAPs (Figs. 1, 2, 3, 4).

### Innovation in evolution

In Figure 5, a proposed crude time line is shown for the evolution of multi-subunit RNAPs. Although modern enzymes bear the scars of evolution, many core protein motifs are strongly conserved for homologous enzymes among the three domains of cellular life (i.e., Figure 4). Considering that LUCA is estimated to have evolved about 3.5 to 3.8 bya, this strong conservation in all cellular life is remarkable. Because of this “crystallization” of core functions since LUCA, many modern enzymes remain conglomerates of motifs that can be traced in evolutionary time by comparison of the relatedness of different modern organisms. From these comparisons, time lines for the evolution and divergence of modern life forms can be estimated. Because LUCA is so ancient and core protein motifs are so well preserved since LUCA, the RNA-protein world is posited to have been a time of far greater evolutionary innovation in generation of novel proteins and core protein motifs. Because the age of the earth (~4.5 billion years) is not much older than LUCA, the span of the RNA-protein world by comparison must have been relatively short. I posit that one reason for rapid evolutionary innovation in the RNA-protein world is that genomes may have been made up of colonies of semi-independent self-replicating RNA elements, so one genome might contain multiple RNAP genes, allowing for greater evolutionary experimentation with core protein functions. LUCA, by contrast, likely depended on a unified but compact DNA genome, in which genes are likely to be much more interdependent, because killing of a single essential gene (i.e., RNAP) potentially kills the entire organism. As with modern eubacteria, furthermore, to optimize replication rates, LUCA may have been strongly selected for a small DNA genome and thus may not have been able to maintain multiple copies of many essential genes. LUCA, therefore, may have sacrificed high evolutionary innovation, as hypothesized for the slower-paced RNA-protein world, for increased metabolic and reproductive efficiency that helped LUCA to out compete and ultimately to destroy most evidence of the RNA-protein world.

**Figure:**
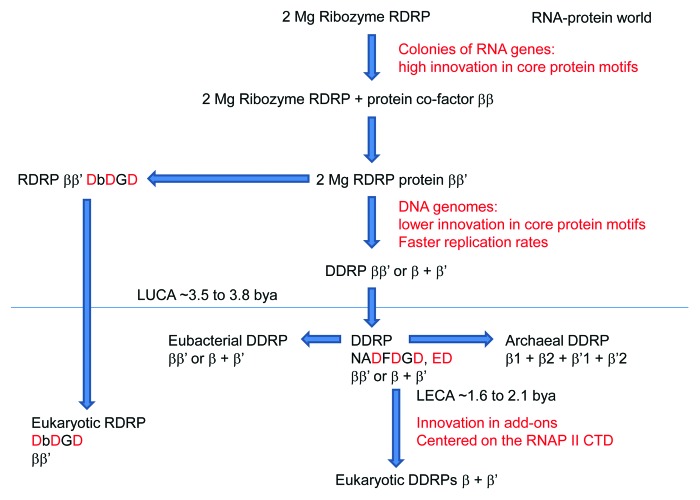
**Figure 5.** A rough timeline and description of the evolution of two DPBB type DDRPs and related RDRPs and proposed mechanisms for innovation in evolution.

In the ancient RNA-protein world, it is thought that self-replicating RNA ribozymes (ribozyme RDRPs) were invaded by a protein cofactor including two DPBB motifs.^4^ These two DPBB motifs evolved into β and β’ types as seen in both RDRPs and DDRPs today (Figs. 2A and 2B). For DDRPs, the β subunit type DPBB was invaded by a SBHM that became the interaction surface for initiation and elongation factors (Fig. 3) required for use of a DNA template.^4^^,^^6^ In this way, DDRPs evolved from RDRPs, prior to LUCA. Since LUCA, DDRPs of the two DPBB type have persisted in eubacteria, archaea and eukarya (including humans). The β and β’ type DPBBs in DDRPs frame the active site, participate in catalysis (i.e., by holding Mg atoms) and may form the core of the motor that drives RNAP translocation in accurate single base steps.

Furthermore, in all cellular life, gene regulation imposes many constraints on evolution of RNAP core protein motifs because changing RNAP activity may alter many dependent and potentially essential processes required for gene expression. In eukaryotes, most evolutionary innovation is expected in RNAP II add-ons rather than within core RNAP motifs. MRNA synthesis by RNAP II specifies protein identity, which gives different cell types unique characteristics. Innovation around RNAP II gene regulation is essential to generate eukaryotic organisms of high complexity, such as animals of highly complex body structure.

At the C-terminal end of its β’ type subunit (named Rpb1), RNAP II has an unusual repeat structure termed the CTD for carboxy terminal domain.^9^^-^^15^ In humans and other vertebrates, the consensus sequence YSPTSPS (Y = tyrosine; S = serine; P = proline; T = threonine) is repeated 52 times (some repeats are non-consensus; see below). The CTD repeat is the focus for high innovation in mRNA regulation in the eukaryotic lineage and has allowed eukarya to develop into myriad complex, multi-cellular organisms. This is the New Testament of evolution of life on earth from the perspective of RNAP structure, function, evolution and associated gene regulation (Fig. 6).^16^

**Figure:**
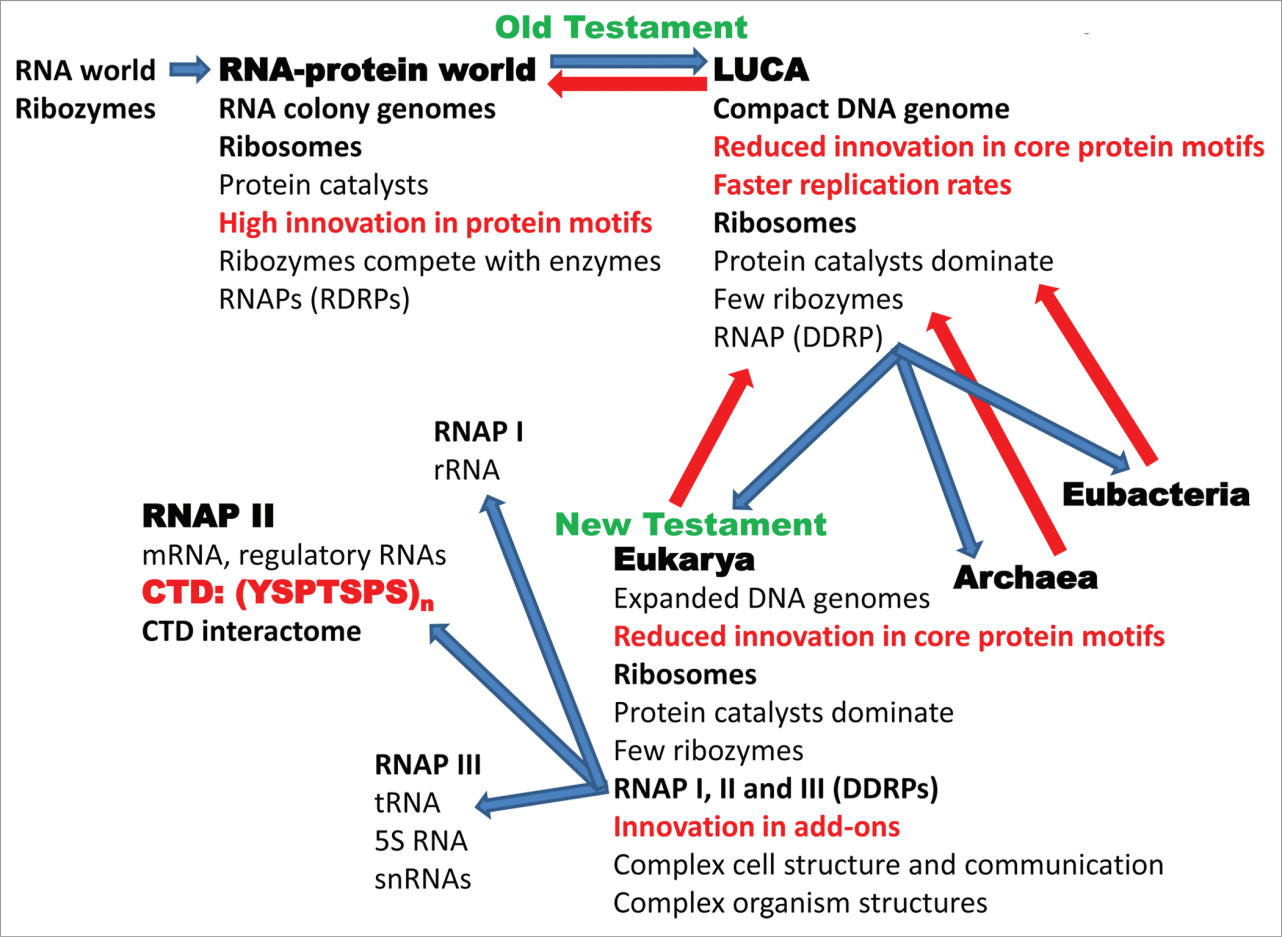
**Figure 6.** The Old and New Testaments of molecular biology describing evolution of multi-subunit RNAPs from the RNA-protein world to LUCA and branching to LECA. The red arrows indicate that LUCA may have eaten the RNA-protein world and that more modern organisms may have devoured and/or out competed LUCA. According to this description, eubacteria and archaea maintain similar features to LUCA. Higher order complexity in eukaryotic gene regulation developed around the CTD of RNAP II and chromatin, and these processes are posited to be strongly co-evolved.

## The New Testament of eukaryotic gene regulatory networks

What makes eukaryotes distinct from mostly single-celled eubacteria and archaea? Specifically, what evolutionary innovations permit eukaryotes to generate high complexity in DNA genome, cell structure, cell communication and organism plan? A hypothesis of this paper is that immense innovation developed around gene regulation, centered on and interacting with the CTD of RNAP II. Innovation confined to mRNA synthesis was crucial to development of organism plans of increasingly higher complexity, and offered the additional benefit of, and probable requirement for, insulating ribosomal, transfer, 5S and regulatory RNAs from innovations in mRNA synthesis.

Yang and Stiller^17^ have recently completed a comprehensive analysis of the evolution of the RNAP II CTD. The CTD appears to be as old as the eukaryotic lineage, so the last eukaryotic common ancestor (LECA; ~1.6 to 2.1 bya) is likely to have had RNAP I, II and III and a multiple heptad (seven amino acid) repeat CTD on RNAP II. Initially, the CTD may have provided a scaffold for binding the spliceosome for co-transcriptional removal of introns from heterogeneous nuclear RNAs.^17^^-^^19^ With time, many additional functions have attached to the CTD scaffold (Fig. 7). All eukaryotes appear to have a CTD or a CTD remnant on RNAP II. In evolution of eukaryotic taxa, the CTD has been repeatedly amplified, partly or completely degenerated in sequence, in some organisms, and, in some cases, re-amplified as a heptad repeat. Just N-terminal to the CTD is a linker sequence that is rich in SP and may represent ancient degeneration of heptad repeat sequences. Interestingly, within the fungi and red algae, increased multi-cellular complexity appears to correlate with a more highly degenerate CTD, indicating that modification and degeneration of CTD repeats can be linked to co-evolution of interacting factors to support specific transcriptional functions and differentiation programs. By contrast, complex animals and plants remain fully dependent on a CTD that includes many consensus heptad repeats, indicating that, in these organisms, although some repeats can degenerate or adapt to specific interactions (as in fungi and red algae), the most complex CTD interactomes also require maintenance of a consensus heptad repeat interaction scaffold.^17^

**Figure:**
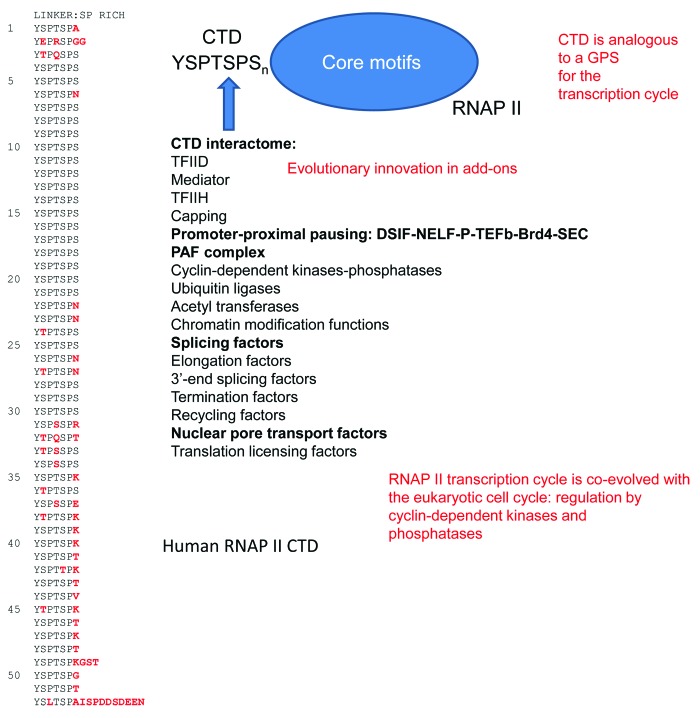
**Figure 7.** The CTD of RNAP II is partly analogous to a GPS for the transcription cycle. The RNAP II transcription cycle, which is centered on the CTD and its interactome, appears co-evolved with the eukaryotic cell cycle. The sequence of the 52 heptad repeat human CTD is shown. Non-consensus heptads are indicated by red letters.

The CTD of RNAP II was a major innovation at the inception of the eukaryote lineage separating the regulation of mRNA synthesis, which in eukaryotes requires highly nuanced regulation, from rRNA synthesis, tRNA synthesis and small regulatory RNA synthesis, which generally require less sophisticated control. Many systems of surprisingly high complexity have evolved around the CTD to modulate RNAP II gene expression including TFIID, SAGA, mediator, TFIIH, COMPASS (Set1 lysine methyltransferase), capping factors, splicing factors, the PAF complex, Set2 complex, 3′-end RNA processing machinery, termination factors, recycling factors and nuclear pore mRNA transport factors (Fig. 7).^13^^-^^15^^,^^20^ Most of these factors conspire with the CTD of RNAP II in carefully regulated interactions. The CTD is heavily modified by phosphorylation/dephosphorylation (and complementing modifications and interactions) involving a cascade of cyclin-dependent kinases and phosphatases, making the transcription cycle analogous to a eukaryotic cell cycle with defined and regulated barriers and check points (Fig. 7).^16^ Modification of the CTD depends on the position of RNAP II in the transcription cycle.

### Promoter-proximal pausing

As a milestone in the eukaryote animal lineage, promoter-proximal pausing of RNAP II is a gene regulation innovation associated with generation of complex body plans and centered on the CTD.^21^^-^^27^ This innovation likely underpins the dramatic Pre-Cambrian and Cambrian Explosion in animal complexity. Animals with highly complex body plans utilize the negative elongation factor NELF complex (NELF subunits A, B, C, D and E), and NELF appears to be absent or not to be fully encoded in the genomes of less structurally complex species, suggesting that NELF components, NELF's recruitment and/or its modification may be key markers for evolution of intricate animal body plans. NELF interacts with the nearly universal elongation factor DSIF (Spt4-Spt5) (Fig. 3) to pause RNAP II near to the promoter. In complex animals, DSIF is converted from a co-repressor with NELF of early pausing by RNAP II to a co-activator and stimulatory elongation factor. DSIF modification occurs through multiple phosphorylations by a highly regulated cyclin-dependent kinase (P-TEFb; positive transcription elongation factor b) that also multiply phosphorylates the RNAP II CTD on Ser2 of the heptad repeat, a modification associated with productive elongation and recruitment of elongation, chromatin modification and termination factors. Animals with complex body patterns (i.e., the fruit fly *Drosophila*) include NELF and utilize promoter-proximal pausing; some animals with less complex body types (i.e., the small nematode roundworm *C. elegans*) lack NELF subunits and do not demonstrate promoter-proximal pausing. From similar logic, innovations such as the notochord in the vertebrate lineage may relate to modifications in P-TEFb recruitment to overcome promoter proximal pausing, such as the Super Elongation Complex (SEC) and/or Brd4, factors implicated in human viral infections (HIV-1→AIDS)^21^^,^^28^^,^^29^ and cancers (mixed lineage leukemias and lymphomas).^30^^,^^31^ The eukaryotic lineage is defined by development of ever more complex, multi-cellular and multi-organ interaction and communication. Organismal complexity must be supported by increasing nuance in regulation of gene expression, much of it involving the CTD, which appears to be the defining characteristic of RNAP II that licenses such specific and complex control of mRNA synthesis in eukaryotes. Because eukaryotes have similar RNAP II transcription cycles and cell cycles, these processes must be co-evolved.^32^ Complex cell signaling, epigenetics and the RNAP II transcription cycle also appear co-evolved, underscoring the importance of the RNAP II CTD in evolution of eukaryote complexity.

### The PAF complex

The PAF complex is a central feature of the RNAP II CTD interactome from yeast to plants to humans. The human PAF complex is implicated in disease^33^ and also drives transcription through nucleosomes in response to transcriptional activators.^34^ In HIV-1 transcription, the role of the SEC in promoter proximal pausing is strongly linked to the PAF complex.^29^ The promoter proximal pausing mechanism of complex animals, therefore, interacts with the PAF complex indicating the central importance of the RNAP II CTD and its interactome in eukaryote evolution. Similarly to less complex animals, plants appear to lack promoter proximal pausing but have highly complex organism plans with organs and vasculature. In plants, the two primary events in the reproductive life cycle are flowering and seed development, and both can be governed by the PAF complex.^35^^-^^39^ The timing of flowering can be controlled by vernalization, which induces flower development after exposure to cold, as in bulb forcing by chilling before planting. In the small mustard *Arabidopsis*, mutations that alter the timing of blooms in response to cold and also the hardening of seeds after flowering are found in multiple genes encoding subunits of the PAF complex, which interacts with the CTD and participates in the initiation, elongation and termination phases of the RNAP II transcription cycle.^33^

### Splicing and complex exon-intron eukaryotic gene structures and genomes

Complex eukaryotic DNA genomes were partly initiated during endosymbiosis of a pro-mitochondrial proteobacterium into an archaeal host.^18^ This event is hypothesized to have unleashed a “catastrophic invasion” of group II introns encoded within the proteobacterium into the archaeal, pre-LECA (“prekaryote”) genome. Because group II introns encode a reverse transcriptase, these elements can insert introns as DNA into a DNA genome.^40^ In response to this genomic explosion, the CTD on RNAP II may initially have evolved to ensure co-transcriptional splicing to remove group II introns that were losing the capacity to self-splice.^17^ Heterogeneous nuclear RNA splicing and the coupled development of complex gene structures including exons and introns are major drivers of eukaryotic genome complexity and evolution. Binding the splicing apparatus to the RNAP II CTD, therefore, provides another powerful example of evolution of eukaryotic gene regulation and mRNA processing embedded in the CTD interactome. In a recent review, Eick and Geyer^13^ discuss the importance of the CTD in evolution of splicing and complex gene structures and regulation in detail.

### Rube Goldberg and eukaryotic transcription

Nuanced gene regulation in the eukaryotic lineage has been developed as a series of seemingly endless add-ons and adaptations: repression, anti-repression and anti-anti-repression, etc. Notably, many or most gene activation mechanisms in eukarya appear to be anti-repression mechanisms. Many or most key evolutionary RNAP II add-ons integrate with the CTD, which can be viewed as somewhat similar to, but somewhat more than, a global positioning system to monitor, locate and regulate progression of RNAP II through and beyond the transcription cycle of pre-initiation complex assembly, initiation, promoter escape, capping, promoter-proximal pausing, elongation, splicing, termination, RNAP II recycling, mRNA folding and decoration, and mRNA export and licensing for translation (Fig. 7).^13^^-^^15^^,^^20^ CTD-mediated control also allows sophisticated communication between the transcription apparatus and chromatin structure, layering a very complex and dynamic landscape on command and control. Many large and seemingly overly complex multi-subunit complexes, including many enzymes for protein modification/de-modification, interact with the CTD, RNAP II and/or chromatin to regulate the processes of gene readout. Such nuance could not develop primarily around sequence-specific DNA binding transcription factors, as in eubacteria (Fig. 8).^6^ Also, eubacteria and archaea utilize only a single RNAP for all RNA synthesis, so these organisms cannot as easily protect ribosomal, transfer and regulatory RNA synthesis from collateral damage resulting from otherwise disruptive modifications in gene expression that affect mRNA synthesis. The eukaryotic RNAP II apparatus and gene regulatory network, therefore, resemble a robustified Rube Goldberg device of endless add-ons, strengthened by many supporting pathways. In the eukaryote line, life is not robust because of elegant design or refinement; life is robust because of functional redundancy in add-ons.

**Figure:**
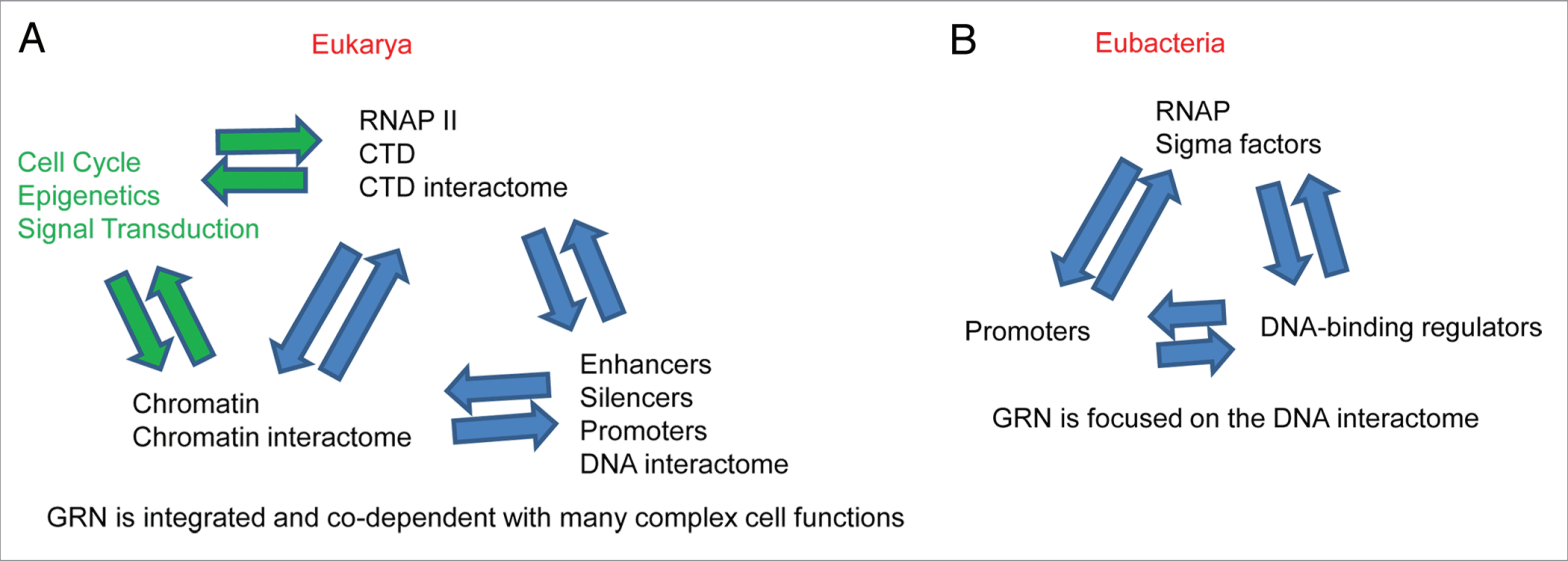
**Figure 8.** Gene regulatory network (GRN) structures (blue arrows) and co-evolved processes (green arrows) for (**A**) eukaryotes and (**B**) eubacteria.

### Co-evolution of eukaryote complexity and gene regulatory networks

Mostly single-celled eubacteria and generally more complex eukarya have characteristic gene regulatory network strategies that likely reflect and license organism complexity (Fig. 8). My opinion is that eukaryote complexity is supported by a distinctly eukaryotic gene regulatory network structure, in which chromatin, the chromatin interactome, the RNAP II CTD and the CTD interactome are defining and interacting components (Fig. 8A). Development of new scaffolds, such as the RNAP II CTD, and mechanisms for DNA maintenance and transcription rendered possible the evolution of mechanisms that could be re-directed to other cell processes. By re-assigning factors to new roles, complexity of the gene regulatory network in eukarya very likely helped drive development of complex epigenetics and signal transduction, which are also now defining features of eukaryotic radiations. Eubacteria, by contrast, because their gene regulatory networks are focused on the DNA interactome (Fig. 8B)^6^ and mostly lack additional dimensions, have much simpler signal transduction mechanisms. The surprising similarities comparing the eukaryotic RNAP II transcription cycle and the eukaryotic cell cycle indicate that these processes are co-evolved.^32^ Eukaryotes require very complex transcriptional control, complex cell cycle control, cell compartmentalization, cell structure, epigenetics and signal transduction. As an example, enclosure of the nucleus in eukarya, relates directly to the RNAP II CTD because nuclear pore transport of mRNA is strongly coupled to the CTD (Fig. 7).^20^ From the standpoint of transcription regulation, eukaryote biological complexity and gene regulatory networks appear strongly co-evolved, mutually interacting and reinforcing (Fig. 8A).

## Evolution vs. special creation

Because only some evolutionary processes can be observed experimentally, “proving” arguments regarding ancient evolution is challenging, but science is much more suited to detailed, accurate and useful description and prediction than “proof.” Also, evolution via natural selection is not nice, and LUCA is strongly suspected of eating the RNA-protein world, limiting the RNA-protein world's availability for current study, just as more modern organisms subsequently may have feasted on or out competed LUCA (Fig. 6). Despite loud current politics, evolution remains an exceptionally powerful concept that describes defining events in the genesis of life on earth in surprising detail and increasingly in concept. Evolution also provides insight into protein evolution, function and dynamics. Here, I tell part of the story of genesis based on early innovations (DPBB and SBHM: a subset of the highly conserved core protein motifs in all multi-subunit RNAPs; Figures 1, 2, 3, 4) that resulted in uninterrupted endurance of multi-subunit RNAPs through billions of years. The story appears to be rooted in the RNA-protein world prior to LUCA, which was ~3.5 to 3.8 bya. In large part, eukarya (beginning ~1.6 to 2.1 bya) appear to have distinguished themselves from eubacteria and archaea through generating increased complexity in mRNA control, complementing development of increasingly complex cell and organism patterns. Here, I allude to some of this complexity without describing it in largely known but potentially excruciating detail.^13^^-^^15^^,^^35^ Within the eukaryotic line, access to varied niches drove evolution to develop hyper-complexity in cell structure, cell communication, organism and animal body plans. At a formative stage, innovation in the eukaryote line required sequestration of mRNA synthesis by RNAP II from other RNA synthesis by RNAPs I and III and subsequently a seemingly endless progression of repression and anti-repression Rube Goldberg controls, most of which interface with the CTD of RNAP II (Fig. 7). It is not clear to this author that theories of special creation or intelligent design as described in the Bible, Koran, Torah or Dhammapada provide as lucid a description of molecular genesis or the panoply of life currently extant on earth. The theory of evolution, by contrast, proves resilient and useful to describe the ancient RNA-protein world, multi-subunit RNAPs and separate radiations of the eubacterial, archaeal and eukaryal lineages. The story of Genesis in the Bible cannot now be modified, but, in principle, as a scientific theory, the story of evolution can be refined or potentially falsified. The expectation among scientists, however, is that the concept of evolution will be extended and remain highly predictive, and, so far, scientists see no limitations to the utility and descriptive capacity of this remarkably powerful and enduring idea. As a dramatic example, Darwin's initial ideas were developed to describe whole organism biology but now apply ever more powerfully to molecular biology and coding, which Darwin, living in the 1800s (1809 to 1882 C.E.), could not have anticipated, making Darwin's seminal hypotheses exceptionally predictive. What scientist of the modern world would not wish to have his/her hypotheses remain predictive and cited for over a hundred years?

### Teaching evolution

Because evolution has become a political football, this complicates teaching. Scientists remain adamant about presenting evolution, without which biology would make no sense, a suspected goal of anti-evolutionists. For one thing, without a conceptual framework, biology seems overwhelmingly complex, and, after more than 100 y of critical and sophisticated challenges to Darwinian philosophy, the most powerful organizing principle in biology remains evolution via natural selection. The current work indicates, however, that underlying the remarkable complexity in biological systems are deeper concepts: that although biological systems naturally generate huge variation and complexity, there may not be very many identifiably distinct “strategies” or milestones in evolution. The splitting of RNAP functions into RNAP I, II and III and extension of the CTD heptad repeat on RNAP II, at the dawn of the eukaryotic lineage, represent major “strategies” and milestones. The RNAP II CTD then became a scaffold for co-evolution of the extensive CTD interactome. Surprisingly, the eukaryotic cell cycle and RNAP II transcription cycle, which is centered on the CTD, resemble one another,^32^ indicating that new “strategies” for development of core biological regulatory cycles may be limited (Fig. 7). By contrast, in eubacteria, there appears to be a limited number of, or limited evolutionary pressure for, strategies to develop new plans for transcriptional regulators, and these limitations on available “strategies” may restrict the capacity of eubacteria to develop highly complex cell types and organism structures (Fig. 8B).^6^ “Strategies” is placed in quotation marks because evolution does not appear to follow pre-planned intelligent design. Rather, successful strategies in evolution become apparent in retrospect, after competing strategies are killed through natural selection. I recommend teaching evolution based in part on evolutionary “strategies” to provide understanding and context and to de-emphasize initially bewildering complexity.

Another concept is that it may be most useful to present evolution based on conservation of protein motifs and protein sequences rather than by concentrating on the description of intact organisms (Figs. 1, 2, 3, 4). Because a convincing phylogenetic tree can readily be constructed based on amino acid sequences and protein motifs, as with RNAP,^5^^,^^41^ it becomes very difficult to deny the conclusion that evolution describes phylogeny. Presenting evolution, I suggest emphasizing genetic coding and molecular evolution of protein sequences and protein motifs, because arguments based on coding prove the most challenging to refute. Flatly stated, there is no rational possibility that protein sequences and motifs can be viewed as a matter of belief. Furthermore, if a student leaves class lacking a “belief” in evolution, at least they may have learned something about molecular coding.

### RNAPs and evolution

I do not mean to imply that the evolution of RNAPs explains all of biology. RNAP, however, provides a rich and coherent lesson because RNAPs are central components in genetic coding and because the evolutionary history of RNAPs extends into the RNA-protein world to LUCA branching to LECA and to the present day, relating a credible story of genesis. Also, gene expression is a core feature of evolutionary advancements, and eukaryotic gene regulation appears to center on the CTD of RNAP II, indicating that the gene regulatory networks of eukaryotes should be understood in significant part through analysis of the extensive CTD interactome (Fig. 7). Because of their clear roles in animal and plant development and human disease, I posit that promoter proximal pausing (SEC, P-TEFb, Brd4, DSIF, NELF; in complex animals) and the PAF complex (in most eukaryotes) may be particularly central in CTD interactome functions in evolution. Because the eukaryotic cell cycle, chromatin epigenetics and signal transduction appear co-evolved with the CTD interactome, I suggest that analysis of the CTD interactome may be a key to understanding eukaryote complexity both including and beyond the roles of the CTD in gene regulation (Fig. 8A). By contrast, the simpler gene regulatory network structure of eubacteria appears less capable of supporting or developing comparable complexity (Fig. 8B). Because the RNAP II CTD appears central to the eukaryotic evolutionary “strategy,” dissection of the RNAP II gene regulatory network is likely to require close attention to the RNAP II CTD and its complex interactome.

## Abbreviations:

AIDS: auto-immune deficiency syndrome
COMPASS: complex proteins associated with Set1
CTD: carboxy terminal domain of RNAP II
DDRP: DNA-dependent RNA polymerase
DPBB: double-psi beta barrel
DSIF: DRB-sensitivity inducing factor
HIV-1: human immunodeficiency virus-type 1
LECA: last eukaryotic common ancestor
LUCA: last universal common ancestor
NELF: negative elongation factor
Nus: N-utilization factor
PAF: polymerase-associating factor
P-TEFb: positive transcription elongation factor b, a cyclin-dependent kinase
RDRP: RNA-dependent RNA polymerase
RNAP: RNA polymerase
SAGA: Spt-Ada-Gcn5 acetyltransferase
SBHM: sandwich barrel hybrid motif
Sc: *Saccharomyces cerevisiae*
SEC: super elongation complex
Spt: suppressor of Ty insertions
TFII: transcription factor for RNAP II
Tt: *Thermus thermophilus*
